# Melatonin ameliorates intervertebral disc degeneration via the potential mechanisms of mitophagy induction and apoptosis inhibition

**DOI:** 10.1111/jcmm.14125

**Published:** 2019-01-04

**Authors:** Yu Chen, Yanqing Wu, Hongxue Shi, Jianle Wang, Zengming Zheng, Jian Chen, Xibang Chen, Zengjie Zhang, Daoliang Xu, Xiangyang Wang, Jian Xiao

**Affiliations:** ^1^ Department of Orthopaedic Surgery The Second Afliated Hospital and Yuying Children’s Hospital of Wenzhou Medical University Wenzhou China; ^2^ School of Pharmacy, Key Laboratory of Biotechnology and Pharmaceutical Engineering Wenzhou Medical University Wenzhou China; ^3^ Institute of Life Sciences Wenzhou University Wenzhou China

**Keywords:** apoptosis, intervertebral disc degeneration, melatonin, mitophagy, oxidative stress

## Abstract

Intervertebral disc degeneration (IDD) is a complicated disease in patients. The pathogenesis of IDD encompasses cellular oxidative stress, mitochondrion dysfunction and apoptosis. Melatonin eliminates oxygen free radicals, regulates mitochondrial homoeostasis and function, stimulates mitophagy and protects against cellular apoptosis. Therefore, we hypothesize that melatonin has beneficial effect on IDD by mitophagy stimulation and inhibition of apoptosis. The effects of melatonin on IDD were investigated in vitro and in vivo. For the former, melatonin diminished cellular apoptosis caused by tert‐butyl hydroperoxide in nucleus pulposus (NP) cells. Mitophagy, as well as its upstream regulator Parkin, was activated by melatonin in both a dose and time‐dependent manner. Mitophagy inhibition by cyclosporine A (CsA) partially eliminated the protective effects of melatonin against NP cell apoptosis, suggesting that mitophagy is involved in the protective effect of melatonin on IDD. In addition, melatonin was demonstrated to preserve the extracellular matrix (ECM) content of Collagen II, Aggrecan and Sox‐9, while inhibiting the expression of matrix degeneration enzymes, including MMP‐13 and ADAMTS‐5. In vivo, our results demonstrated that melatonin treatment ameliorated IDD in a puncture‐induced rat model. To conclude, our results suggested that melatonin protected NP cells against apoptosis via mitophagy induction and ameliorated disc degeneration, providing the potential therapy for IDD.

## 1 INTRODUCTION

Intervertebral disc (IVD) degeneration (IDD) is a widely known contributor to lower back pain (LBP), and is a prevalent musculoskeletal disorder that results in a massive socioeconomic burden worldwide^1^, ^2^, ^3^. However, until very recently, no specific therapies for IDD existed.

The intervertebral disc is composed of a gelatinous inner core, the nucleus pulposus (NP), and tough outer rings and the annulus fibrosus (AF), which are sandwiched with cartilage endplates between the two adjacent vertebrae.^4^ The main functional composition of discs is gelatinous NP, which allows the discs to confront diverse mechanical impacts, whereas the tough AF forms a circular ring structure to support the NP.^5^ Due to the lack of vascularization in the IVD, the main way of transportation of nutrients, oxygen, metabolic products and water for the inner disc cells is their diffusion from capillaries through the cartilage endplates to the cells of the disc.^6^ NP is critically important for maintaining the physiological function of discs. Nucleus pulposus (NP) cells are the main type of cells resident in the NP. They produce extracellular matrix (ECM), such as collagen I, collagen II and proteoglycan, which are the main components of gelatinous tissues of the NP.^7^ Excessive apoptosis of NP cells can trigger IDD, and has been proposed as a therapeutic target for IDD.^8^

Reactive oxygen species (ROS) are a potent proapoptotic factor for human NP cells, and are considered essential mediators of the occurrence and progression of IDD.^9^, ^10^ ROS mediates the proapoptotic effects of various external stimuli on disc cells, including mechanical loading, nutrition deprivation and pro‐inflammatory cytokines.^11^, ^12^ These stimuli can cause ROS overproduction and decrease mitochondrial membrane potential (Δψm), which results in mitochondrion dysfunction in NP cells.^10^, ^13^ Furthermore, mitochondrion dysfunction can enhance ROS production in disc cells by forming a positive feedback loop.^14^, ^15^ Therefore, an overproduction of ROS can induce NP cell apoptosis through the mitochondrial apoptosis pathway.

Mitophagy is a special type of autophagy that maintains mitochondrial homoeostasis by eliminating damaged mitochondria and reducing cellular stress caused by aberrant oxidative bursts.^16^ Basal levels of mitophagy can maintain cellular homoeostasis and protect cells against dysfunctional mitochondria. During stress, a concomitant activation of mitophagy and apoptosis is triggered, whereby enhanced mitophagy can promote survival by eliminating damaged mitochondria.^17^ Mitophagy has been associated with mitochondrion dysfunction and apoptosis in the pathological process of several diseases, including Parkinson's disease,^18^ heart disease^19^ and liver fibrosis.^20^ Given that oxidative stress, mitochondrial dysfunction and apoptosis mediate the pathogenesis of IDD, therefore, induction of mitophagy may protect against the early stages of IDD.

Melatonin (N‐acetyl‐5‐methoxytryptamine) is an endogenous molecule released from the pineal gland, and has been studied in several diseases due to its following properties: low toxicity; soluble in both aqueous and organic phases; potent free radical scavenger; influence on mitochondrial homoeostasis and functioning.^21^, ^22^, ^23^ Recently, melatonin has been proven to counteract oxidative stress, inflammatory responses and apoptosis in experimental osteoarthritis models, which have similar pathological processes with IDD.^24^, ^25^, ^26^, ^27^ Moreover, accumulating studies have demonstrated that melatonin may enhance mitophagy in several tissues, including the brain^28^ and liver,^20^ but not yet in NP cells. Therefore, we hypothesized that melatonin can ameliorate mitochondrial dysfunction and apoptosis induced by oxidative stress via mitophagy induction in NP cells and preventive effects on IDD.

In this study, tert‐butyl hydroperoxide (TBHP) was applied to induce oxidative stress, which could mimic the pathological mechanisms of mitochondrial dysfunction and apoptosis in NP cells. The protective effects of melatonin on apoptosis and mitochondrial dysfunction, as well its mechanisms, were investigated in NP cells exhibiting oxidative stress. Finally, the therapeutic potential of melatonin in a puncture‐induced rat IDD model was evaluated.

## 2 MATERIALS AND METHODS

### 2.1 Ethics statement

All surgical interventions, treatments and postoperative animal care procedures were conducted to comply with the Animal Care and Use Committee of Wenzhou Medical University (wydw2014‐0129).

### 2.2 Reagents and antibodies

Melatonin, TBHP and type II collagenases were acquired from Sigma‐Aldrich (St Louis, MO). The primary antibodies of Collagen II, Aggrecan, Sox‐9, MMP‐13, ADAMTS‐5, Bax, Bcl‐2, Cytochrome C, P62, PINK1, Parkin, GAPDH and SOD2 were obtained from Abcam (Cambridge, MA, USA). The LC‐3, Beclin‐1 and Cleaved‐caspase 3 antibodies were acquired from CST (Beverly, MA). The FITC‐labeled and horseradish peroxidase‐labeled secondary antibodies were ordered from Bioworld (MN, USA). The 4', 6‐diamidino‐2‐phenylindole (DAPI) was acquired from Beyotime (Shanghai, China). The cell culture reagents were acquired from Gibco (Grand Island, NY).

### 2.3 Primary nucleus pulposus cell isolation

Rat NP cells were isolated according to previous reports^29^. Forty male Sprague–Dawley rats (150‐200 g) were euthanized with an overdose of 10% chloral hydrate. The lumbar segments of backbone were extracted integrally under sterile conditions and lumbar discs were collected. Gelatinous NP tissues were separated from lumbar discs with a dissecting microscope and digested in 0.25% trypsin and 0.1% type II collagenase for approximately 4 hours at 37°C. After centrifugation at 1000 rpm for 5 minutes, the supernatant was deserted, and the sediment was resuspended in completed DMEM/F12 (1:1) medium supplemented with 10% foetal bovine serum (FBS) and 1% antibiotics (penicillin/streptomycin). Finally, the resuspension was transferred into a culture flask and cultured in an incubator containing 5% CO_2_ at 37°C. Cells from passages 1 to 3 were used for our experiment.

### 2.4 NP cell treatment

To observe NP cells in a concentration‐dependent manner, different concentrations of TBHP (0, 50, 100, 200, 400 and 600 μmol/L) were included into the culture medium of NP cells for 12 hours before checking cell viability. To observe NP cells in a time‐dependent manner, NP cells were treated with 100 μmol/L TBHP at these time intervals: 0, 2, 4, 6, 8 and 12 hours. To evaluate the cell cytotoxicity of melatonin, cells were incubated with increasing concentrations of melatonin (0.1‐10000 μmol/L) for 24 hours. To examine the protective effects of melatonin, NP cells were pretreated with various concentrations of melatonin (0.1, 1, 10 and 100 μmol/L) for 12 hours, followed by TBHP stimulation (100 μmol/L) for 4 hours. To investigate the role of mitophagy in melatonin‐induced cell protection, NP cells were pretreated with 1 μmol/L CyclosporinA (CsA, Solarbio, Beijing, China) for 1 hour before melatonin administration.

### 2.5 Cell viability assay

Cell viability assay was performed by using cell counting kit‐8 (CCK‐8; Dojindo Co, Kumamoto, Japan) according to manufacturer instructions. NP cells were seeded onto 96‐well plates (5000/well) and incubated in DMEM/F12 with 10% FBS and 1% antibiotics at 37°C for 24 hours. Next, cells were treated with TBHP or/and melatonin as described above. The cells were then washed with phosphate‐buffered saline (PBS) and incubated with CCK‐8 solution at 37°C for 1 hour. Finally, well absorbance was calculated at 450 nm by using a micro‐plate reader.

### 2.6 siRNA transfection

To inhibit Parkin expression, NP Cells (70% confluence) were treated with Parkin small interfering RNAs (siRNAs) (Bioneer, Daejeon, South Korea) according to manufacturer instructions. Transfection of NP cells by negative control siRNA or Parkin siRNA was achieved with Lipofectamine 2000 (Thermo Fisher) according to manufacturer protocols. After transfection, cells were further treated as described above, and used for subsequent analysis.

### 2.7 Transmission electron microscopy

NP cells were fixed in 2.5% glutaraldehyde for 12 hours, and post‐fixed in 2% osmium tetroxide for 1 hour, and stained with 2% uranyl acetate for another 1 hour. Afterwards, cells were dehydrated in acetone of ascending gradient, embedded into araldite and sliced into semi‐thin sections. The sections were subsequently stained with toluidine blue to locate cells recorded with a transmission electron microscope (Hitachi, Tokyo, Japan).

### 2.8 Western blot assay

An ice‐cold radio‐immuneprecipitation assay (RIPA) buffer was used to lyse cells with supplemental proteinase inhibitors. Approximately 60 μg protein samples were isolated using sodium dodecyl sulfate‐polyacrylamide gel electrophoresis (SDS‐PAGE) and transported to a polyvinylidenedifluoride (PVDF) membrane (BIO‐RAD, Hercules, CA). After blocking with 5% skim milk for 1.5 hours at room temperature, membranes were incubated in TBST at 4°C overnight with the following primary antibodies: Collagen II (1:1000), Aggrecan (1:200), Sox‐9 (1:1000), MMP‐13 (1:1000), ADAMTS‐5 (1:1000), Bax (1:1000) and Bcl‐2 (1:500), Cytochrome C (1:500), P62 (1:1000), PINK1 (1:1000), Parkin (1:1000), GAPDH (1:10000), LC‐3 (1:1000), Beclin‐1 (1:1000), SOD2 (1:500) and Cleaved‐caspase3 (1:000). After being washed three times with TBST, membranes were incubated with respective secondary antibodies (1:5000) for 1 hour at room temperature. Bands and band densities were detected by using the ChemiDicTM XRS+Imaging System (Bio‐Rad Laboratories) and Image Lab 3.0 software (Bio‐Rad), respectively.

### 2.9 Immunofluorescence

A 6‐well plate was utilized to seed NP cells, with 3 × 10^5^ cells/mL per well. The cells were then incubated for 24 hours. After treatment, cells were fixed for 10 minutes using 4% paraformaldehyde and inhibited with 5% BSA in PBS containing 0.1% Triton X‐100 in a 37°C oven for 30 minutes. Slices were incubated with primary antibodies against LC3 (1:200), TOMM20 (1:400), collagen II (1:200), or MMP‐13 (1:400) at 4°C overnight. The day after, slices were rinsed with PBS and incubated with fluorescent Alexa 568, 647 donkey anti‐mouse/rabbit or 488 goat anti‐mouse/rabbit secondary antibodies (1:1000) for 1 hour in a 37°C oven. The nuclei were labelled with DAPI, and images were captured by using fluorescence (Olympus Inc, Tokyo, Japan) and Nikon ECLIPSE Ti microscopes (Nikon, Japan).

### 2.10 Mitochondrial membrane potential assay (JC‐1)

Following the treatment, NP cells were stained with 10 µmol/L of JC‐1 according to manufacturer protocols (Beyotime, Shanghai, China). The cells were then rinsed twice with medium and imaged under a fluorescence microscope (Nikon, Japan).

### 2.11 Intracellular ATP and ROS assay

Intracellular ATP and ROS levels were determined by using the ATP Assay and Reactive Oxygen Species Assay Kits according to manufacturer instructions (Beyotime, Shanghai, China), respectively.

### 2.12 TUNEL staining

DNA fragmentation was detected by using an in‐situ Cell Death Detection Kit (Roche, South San Francisco, CA). After being fixed with 4% paraformaldehyde for 1 hour, cells were incubated with 3% H_2_O_2_ and 0.1% Triton X‐100 for 10 minutes. Cells were then washed with PBS, and co‐stained with TUNEL inspection fluid and DAPI. Three random microscopic fields per slide were observed under a fluorescence microscope (Olympus Inc, Tokyo, Japan).

### 2.13 Surgical procedure

A total of 48 male 8‐week‐old Sprague Dawley rats were utilized during in vivo experimentation. They were randomly separated into three groups (control group, IDD group and Melatonin group) and anaesthetized by intraperitoneal injection of 10% chloral hydrate (3.6 mL/kg). The IDD group and melatonin group underwent the following operation according to published papers.^30^ The experimental level rat tail disc (Co7/8) was observed by digital palpation to be located on the coccygeal vertebrae, which was further established by trial radiograph. Needles (27 G, about 4 mm in length) were utilized to puncture the annulus fibrosus though the tail skin, perpendicularly. All needles were rotated 360° and kept in place for 1 minute. After surgery, the melatonin group was intraperitoneally injected with melatonin (10 mg/kg/day), which was dissolved in absolute ethanol and further diluted in 0.9% NaCl solution, while the IDD group accepted an equal amount of ethanol and saline solution. Daily injections started on the day of operation, and continued until the rats were sacrificed. All animals were given free unrestricted weight bearing and activity, and were monitored every day to ensure their well‐being.

### 2.14 Magnetic resonance imaging method

Magnetic resonance imaging was conducted with a 3.0 T clinical magnet (Philips Intera Achieva 3.0MR) on the rats at 0, 4 and 8 weeks after operation. Sagittal T2‐weighted images were taken to evaluate signal and structural differences of the discs. T2‐weighted segments in the sagittal plane were acquired in the following settings: (a) fast‐spin echo sequence with a time to repetition (TR) of 5400 ms and time to echo (TE) of 920 ms; (b) 320 (h) 9 256 (v) matrix; (c) 260 field of view; 4 excitations. Segment thickness was 2 mm with a 0 mm gap. MRIs were assessed by another blinded orthopedic researcher using the following classification of intervertebral disk degeneration^16^: one point = Grade I, two points = Grade II, three points = Grade III and four points = Grade IV.

### 2.15 Histopathologic analysis

After MRI examination, rats were euthanized by intraperitoneal administration of 10% chloral hydrate and caudal vertebras, including experimental segment of discs, were extracted. The specimens were then fixed in 4% paraformaldehyde for 24 hours, decalcified in neutral 10% (v/v) EDTA solution for one month, embedded in paraffin and sectioned (5 µm) along the midsagittal plane. To assess disc condition, the cellularity and morphology of NPs were evaluated in a blinded manner according to the grading scale as explained previously.^17^ The histologic score was graded at 5 for normal discs, 6‐11 for moderately degenerated discs, and 12‐14 for severely degenerated discs.

### 2.16 Statistical analysis

For in vitro studies, each experiment was performed in triplicate. The data were expressed as the Mean ± SD. Statistical analyses were conducted by using SPSS statistical software program 18.0. Differences among the experimental groups were identified by one‐way analysis of variance (ANOVA) and Tukey's test. Nonparametric data (Pfirrmann grading) were examined by using the Kruskal–Wallis H‐test. A *P*‐value of (*P* < 0.05) was considered statistically significant.

## 3 RESULTS

### 3.1 Melatonin treatment inhibits apoptosis and enhances synthesis of ECM component

**Figure 1:**
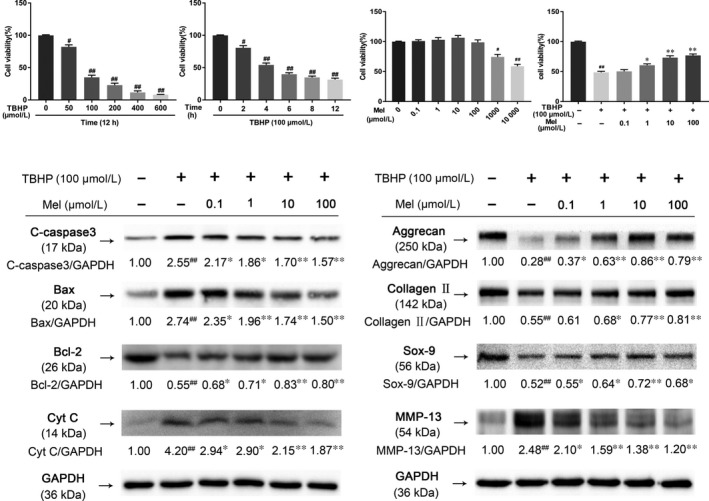
Melatonin treatment decreases apoptosis of NP cells and inhibits degradation of ECM component. (A&B) Effects of TBHP on cell viability detected by Cell Counting Kit‐8 (CCK‐8). (C) Cell Counting Kit‐8 (CCK‐8) results of NP cells treated with different concentrations of melatonin for 24 hours (D) Effect of melatonin on TBHP‐induced cytotoxicity. (E) Protein content of Bax, Bcl‐2, Cytochrome C and Cleaved‐caspase3 of NP cells treated with TBHP and TBHP plus melatonin. (F) Protein content of Aggrecan, Collagen II, Sox‐9 and MMP‐13 of NP cells treated with TBHP and TBHP plus melatonin. The data in the figures represent the averages ±SD ##*P* < 0.01, #*P* < 0.05, vs Control group, **P < 0.01, *P < 0.05, vs TBHP, n = 3

Initially, the cytotoxic effects of TBHP on NP cells were evaluated by using the CCK‐8 assay. As shown in Figure 1A,B, TBHP treatment was observed to reduce cell viability in a dose‐ and time‐dependent manner. TBHP exposure at a concentration exceed 100 μmol/L for 12 hours and TBHP (100 μmol/L) treatment for 4 hours or longer significantly reduced cell viability. Melatonin administration was not observed to show obvious cytotoxicity to NP cells after 24 hours at concentrations more than 100 μmol/L (Figure 1C). Therefore, the concentration of TBHP at 100 μmol/L and melatonin at 0.1, 1, 10 and 100 μmol/L were utilized in our following studies.

Melatonin pretreatment significantly protected against TBHP‐induced cell death (Figure 1D). The western blotting results showed that stimulation of TBHP enormously up‐regulated the expression of pro‐apoptotic protein (Cleaved‐caspase3, Cytochrome C and Bax) and decreased the expression of anti‐apoptotic protein (Bcl‐2). Whereas pretreatment of melatonin reversed TBHP‐induced pro‐apoptotic and anti‐apoptotic protein changes in a dose‐dependent manner (Figure 1E).

Next, the homoeostasis of ECM was evaluated by measuring the expression of ECM major proteins (Collagen II and Aggrecan) and ECM degradation proteinase (MMP‐13). TBHP treatment significantly inhibited Collagen II and Aggrecan contents, as well as Sox‐9 transcription, while increasing the expression of MMP‐13 (Figure 1F). However, administration of melatonin attenuated the loss of Collagen II, Aggrecan, Sox‐9 and the increase of MMP‐13 caused by TBHP treatment.

### 3.2 Melatonin activates parkin and induces mitophagy

**Figure 2:**
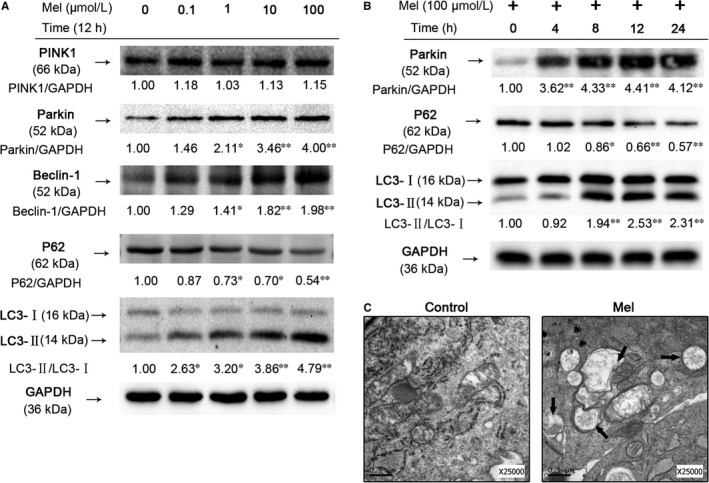
Melatonin treatment induces mitophagy in NP cells. (A) Protein content of PINK1, Parkin, Beclin‐1, P62 and LC3 in NP cells incubated with 0, 0.1, 1, 10 or 100 μmol/L melatonin for 12 hours (B) Protein content of Parkin, P62 and LC3 in NP cells treated with 100 μmol/L melatonin for 0, 4, 8, 12 or 24 hours (C) Mitophagosomes were detected by transmission electron microscopy (×25000) in NP cells. (Black arrow: mitophagosome). The data in the figures represent the averages ±SD **P < 0.01, *P < 0.05, vs Control group, n = 3

The ratio of microtubule‐associated protein 1 light chain 3 II/I (LC3‐II/I), P62, Beclin‐1, PINK1 and Parkin were regarded as indicators of mitophagy induction, and were detected by western blotting. As shown in Figure 2A, the ratio of LC3‐II/LC3‐I, as well as the expression of Parkin and Beclin‐1 were up‐regulated after melatonin treatment for 12 hours in a dose‐dependent manner, whereas the levels of P62 were decreased. No marked differences in the expression of PINK1 were observed between groups. Parkin, LC3‐II/LC3‐I ratio and P62 were then determined at different time points after melatonin treatment (100 μmol/L). Interestingly, Parkin protein levels were significantly upregulated after 4 hours of treatment, but notably changes of the LC3‐II/LC3‐I ratio and P62 were not observed until 4 hours later, suggesting a consistently increased Parkin‐dependent mitophagy induction (Figure 2B). Mitophagy induction was confirmed with transmission electron microscopy (TEM). The melatonin‐treated cells exhibited higher numbers of autophagic vacuoles ingesting mitochondria compared to the control group (Figure 2C).

### 3.3 Inhibition of parkin significantly attenuates melatonin‐induced mitophagy

**Figure 3:**
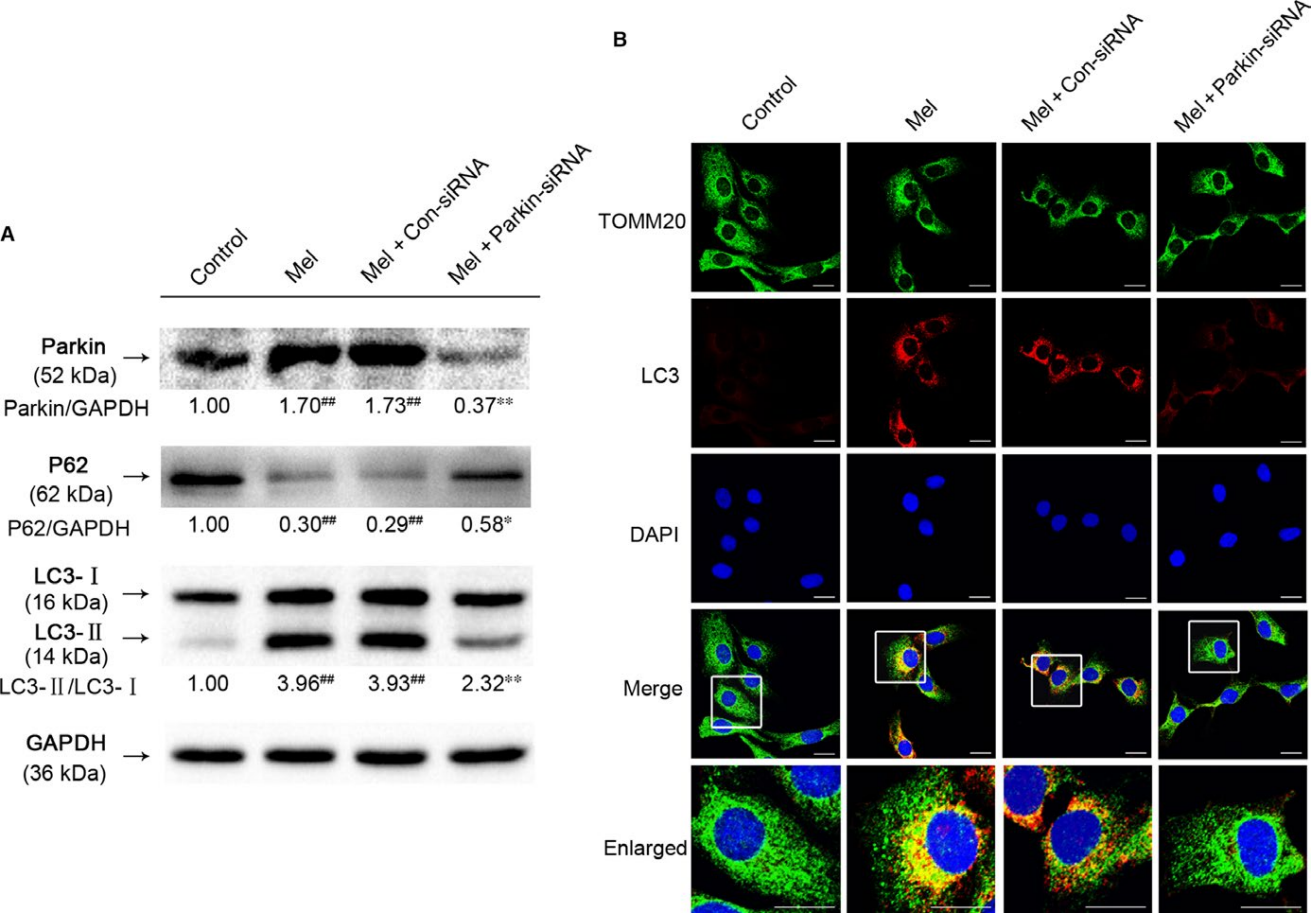
Inhibition of Parkin by siRNA significantly attenuates melatonin‐induced mitophagy in NP cells. Cells were transfected with negative control siRNA (con‐siRNA) or Parkin‐siRNA before receiving melatonin (100 μmol/L). (A) The protein expression of Parkin, P62 and LC3 in the NP cells as treated above. (B) Double immunofluorescence of LC3 protein and TOMM20 protein in the NP cells. (Green signal represents TOMM20, red signal represents LC‐3, scale bar: 25 μm). The data in the figures represent the averages ±SD ##*P* < 0.01, #*P* < 0.05, vs Control group, **P < 0.01, *P < 0.05, vs Mel+Con‐siRNA, n = 3

To further evaluate the role of Parkin in melatonin‐induced mitophagy, NP cells were transfected with Parkin‐siRNA before melatonin treatment. The inhibitory effects of small interfering RNA (siRNA) on melatonin‐induced Parkin activation are shown in Figure 3A. Compared to melatonin and negative control siRNA treatment, Parkin‐siRNA administration markedly reduced Parkin expression and the ratio of LC3‐II/LC3‐I induced by melatonin administration. Furthermore, the reduction of P62 induced by melatonin was reversed in the Parkin‐siRNA group. These results were further confirmed by the co‐localization of LC3 with the mitochondria marker TOMM20, which evidenced mitophagosomes formation. (Figure 3C). Therefore, these results suggested that activation of mitophagy induced by melatonin was dependent on Parkin.

### 3.4 Co‐regulation of mitophagy by TBHP AND melatonin treatment

**Figure 4:**
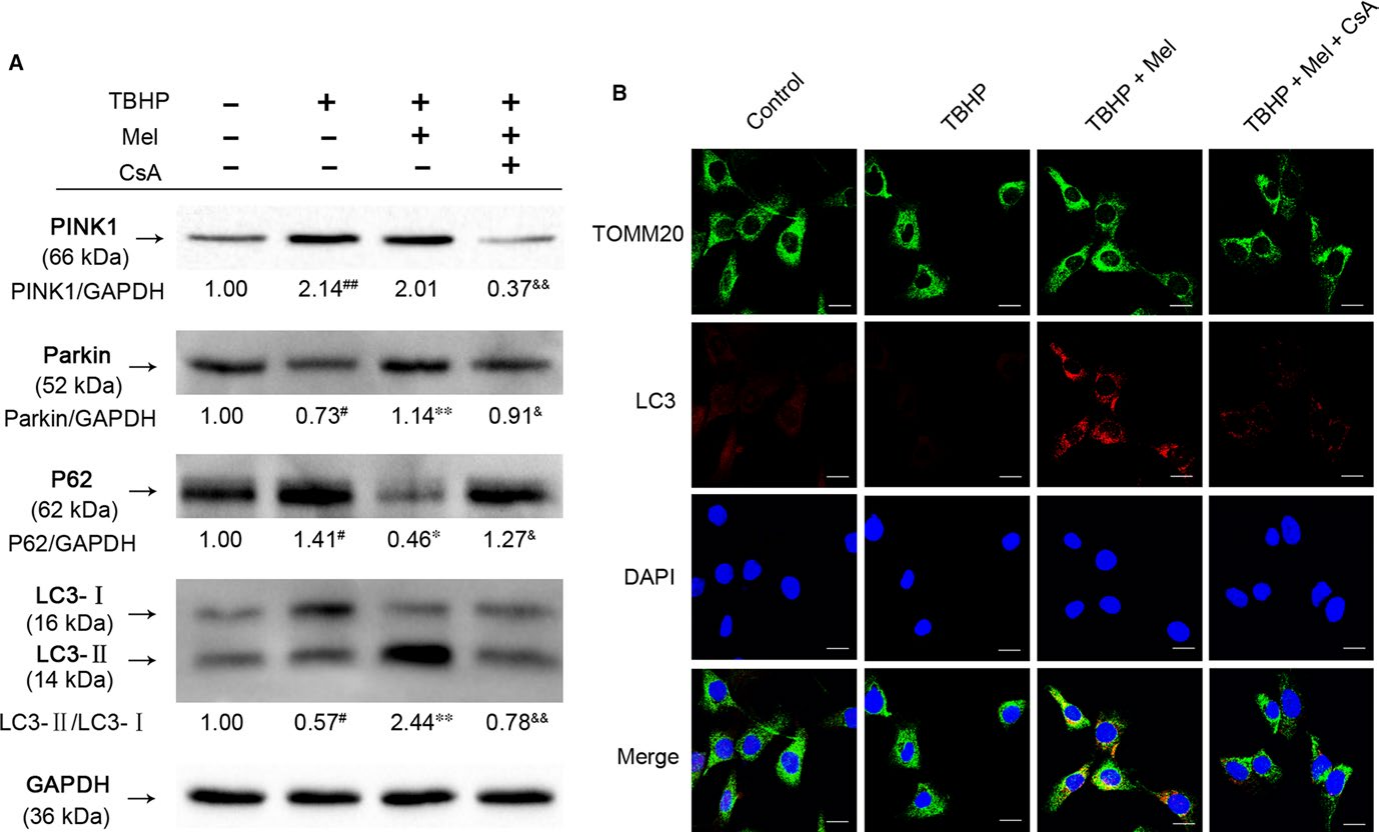
TBHP and CsA regulate mitophagy in NP cells. The NP cells were untreated (DMEM/F12(1:1) 10%FBS), or treated with TBHP alone, or treated with melatonin (100 μmol/L) and TBHP, or treated with TBHP and melatonin (100 μmol/L) combined with CsA (1 μmol/L). (A) The protein expression of PINK1, Parkin, P62 and LC‐3 in the NP cells as treated above. (B) Double immunofluorescence of LC3 protein and TOMM20 protein in NP cells. (Green signal represents TOMM20, red signal represents LC‐3, scale bar: 25 μm). The data in the figures represent the averages ±SD ##*P* < 0.01, #*P* < 0.05, vs Control group, **P < 0.01, *P < 0.05, vs TBHP, &&*P* < 0.01, &*P* < 0.05, vs TBHP+Mel, n = 3

As shown in Figure 4A, TBHP treatment alone increased the protein levels of PINK1, indicating mitochondrial damages in NP cells. Meanwhile, TBHP treatment also reduced the expression of Parkin and caused P62 accumulation, suggesting that TBHP blocked mitophagy induction. However, administration of melatonin significantly attenuated TBHP‐reduced mitophagy activation in NP cells, which was reflected by the increased expression of Parkin and the ratio of LC3‐II/LC3‐I, and the inhibition of P62 accumulation. To inhibit melatonin‐induced mitophagy induction, CyclosporinA (CsA), a mitophagy inhibitor,^31^, ^32^ was utilized. The pretreatment of CsA (1 μmol/L) was observed to cause significant decreases of PINK1，Parkin and the ratio of LC3‐II/LC3‐I, while increasing P62 accumulation compared to the melatonin group. These results were further confirmed by immunofluorescence analysis (Figure 4B). Therefore, these data indicated that TBHP suppressed mitophagy activity, while melatonin effectively reversed TBHP‐reduced mitophagy in NP cells, which can be inhibited by the mitophagy inhibitor CsA.

### 3.5 Melatonin attenuates TBHP‐induced cellular apoptosis VIA MITOPHAGY INDUCTION

**Figure 5:**
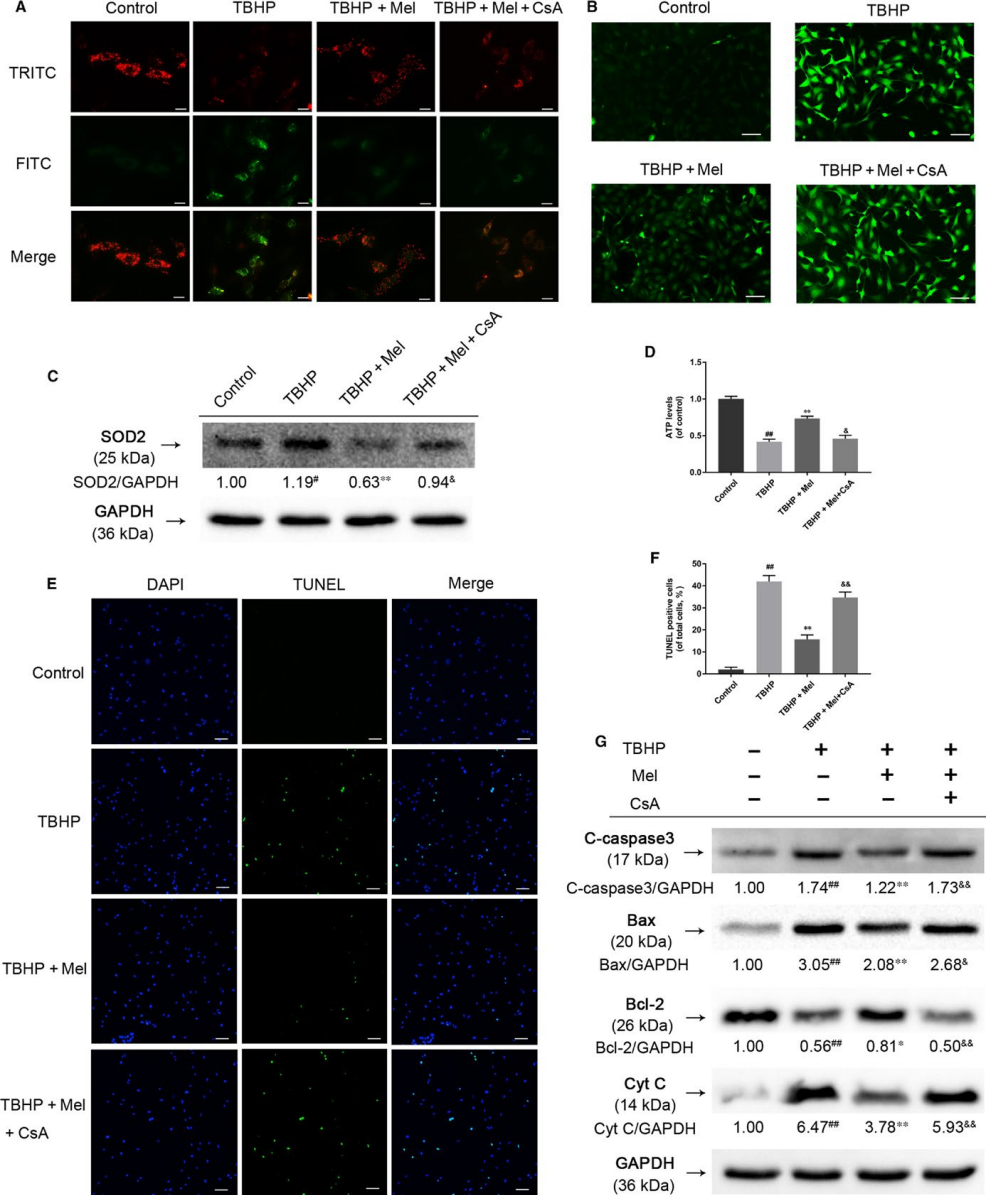
Melatonin treatment attenuates mitochondrion dysfunction and reduces NP cell apoptosis under oxidative stress. (A) Mitochondrial membrane potential in cells was measured by JC‐1 staining. Scale bar: 25 μm. TRITC represents the normal Δψm, FITC represents the collapse of Δψm. (B) Images of DCFH‐DA fluorescence was taken after TBHP stimulation to evaluate the intracellular ROS levels. Scale bars: 50 μm. (C) The protein expression of SOD2 in NP cells treated as above. The data in the figures represent the averages ±SD ##*P* < 0.01, #*P* < 0.05, vs Control, **P < 0.01, *P < 0.05, vs TBHP, &&*P* < 0.01, &*P* < 0.05, vs TBHP+Mel, n = 3. (D) Cellular ATP levels of NP cells were measured after the treatment described above. (E&F) TUNEL assay was performed in NP cells as treated above. Scale bar: 100 μm. (F) The protein expression of Cleaved‐caspase3, Bax, Bcl‐2 and Cytochrome C in NP cells treated as above. The data in the figures represent the averages ±SD ##*P* < 0.01, #*P* < 0.05, vs Control group, **P < 0.01, *P < 0.05, vs TBHP, &&*P* < 0.01, &*P* < 0.05, vs TBHP+Mel, n = 3

To investigate whether mitophagy induction was involved in melatonin‐induced protective effects against TBHP‐induced apoptosis, NP cells were pretreated with mitophagy inhibitor CsA before melatonin administration. Mitochondrial depolarization occurred in TBHP‐treated cells, and was indicated by decreased mitochondrial membrane potential (Δψm) (Figure 5A). While the Δψm was recovered by melatonin treatment compared to the TBHP‐treatment group, the preservation of Δψm failed while the cells were pretreated with both melatonin and CsA. While increased intracellular ROS production induced by TBHP was attenuated by melatonin administration, CsA pretreatment was observed to reverse these effects (Figure 5B). The intracellular ROS level was also reflected by the immunoblot result of SOD2 (Figure 5C), which is a representative antioxidant enzyme located in the mitochondrial matrix. The change of cellular ATP level was consistent with the Δψm results (Figure 5D). Meanwhile, the TUNEL assay results demonstrated a marked increase in the number of apoptotic cells in TBHP treatment group, and melatonin was observed to reverse TBHP‐induced cell apoptosis. Inhibition of mitophagy by CsA administration blocked the protective effects of melatonin (Figure 5E&F). Consistent with TUNEL assay data, TBHP treatment up‐regulated pro‐apoptotic protein (Bax, cleaved‐caspase3 and Cytochrome C) expression, decreased anti‐apoptotic protein (Bcl‐2) expression and melatonin pretreatment attenuated these effects. The effects of melatonin on reducing pro‐apoptotic proteins and increasing anti‐apoptotic proteins were reversed after mitophagy inhibition (Figure 5G). Therefore, these results indicated that the protective effects of melatonin against cellular apoptosis were caused by the stimulation of mitophagy.

### 3.6 Mitophagy induction is necessary for melatonin against ECM degeneration

**Figure 6:**
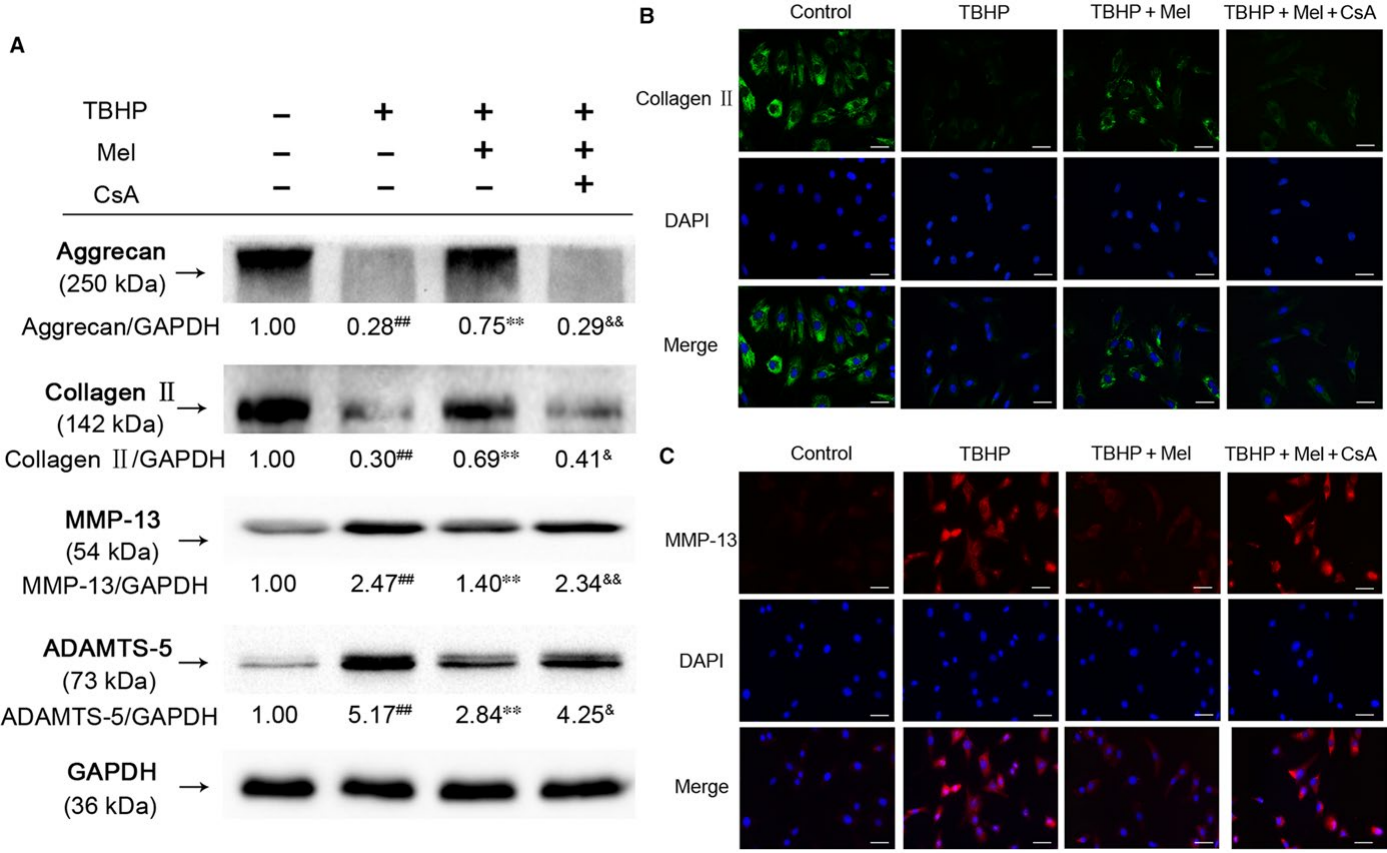
Effects of melatonin against ECM degeneration of NP cells induced by oxidative stress. (A)The protein expression of Collagen II, Aggrecan, MMP‐13 and ADAMTS‐5 were measured by western blot assay. (B&C) The representative collagen‐II and MMP‐13 were detected by the immunofluorescence combined with DAPI staining for nuclei (Green signal represents Collagen II, red signal represents MMP‐13, scale bar: 25 μm). The data in the figures represent the averages ±SD ##*P* < 0.01, #*P* < 0.05, vs Control group, **P < 0.01, *P < 0.05, vs TBHP, &&*P* < 0.01, &*P* < 0.05, vs TBHP+Mel, n = 3

The Western blot results showed that TBHP treatment markedly reduced Collagen II and Aggrecan protein levels, and increased the expression of ECM degradation enzymes MMP‐13 and ADAMTS‐5. The effects of TBHP on ECM were attenuated by melatonin pretreatment. To explore the role of mitophagy in melatonin against ECM degradation, the mitophagy inhibitor CsA was applied before melatonin treatment. The results demonstrated that protective effects of melatonin against TBHP‐induced ECM degradation were blocked with CsA pretreatment (Figure 6A). The expression of collagen‐II and MMP‐13 was also investigated by immunofluorescence analysis (Figure 6B&C), and these results were congruent with the Western blot results. Therefore, these results suggested that mitophagy induction is necessary for melatonin against ECM degeneration induced by TBHP.

### 3.7 Melatonin ameliorates disc degeneration in a rat model of IDD

**Figure 7:**
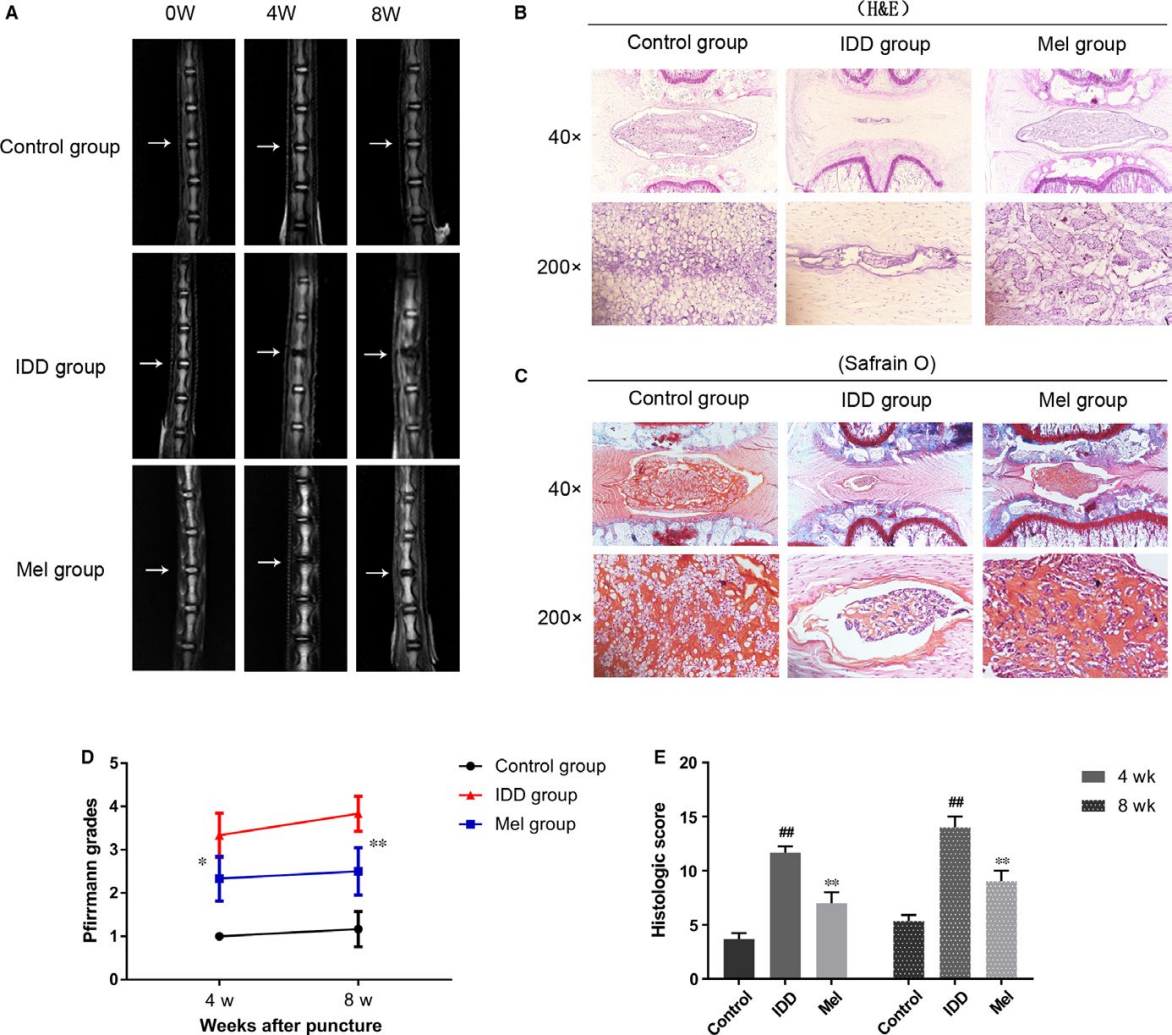
Melatonin treatment amiliorates rat IDD in vivo. (A) T2‐weighted MRI of a rat tail with a needle‐punctured disc at 4 and 8 weeks post surgery (white arrows). (B) Representative H&E staining of disc samples from different experimental groups at 4 and 8 weeks post surgery. (C) Representative Safrain‐O of disc samples from different experimental groups at 4 and 8 weeks post surgery. (D) The Pfirrmann MRI grade scores in three groups at week 4 and week 8. (E) The histological grades evaluated at week 4 and week 8 in three groups. The data in the figures represent the averages ±SD ##*P* < 0.01, #*P* < 0.05, vs Control group, **P < 0.01, *P < 0.05, vs IDD group, n = 6

To evaluate the therapeutic effects of melatonin on degenerative rat discs, magnetic resonance imaging (MRI) and Pfirrmann MRI grade scores were taken at 4 and 8 weeks after puncture. The melatonin treated group was observed to demonstrate higher T2‐weighted signal intensities than the IDD group both at 4 and 8 weeks (Figure 7A). Moreover, the Pfirrmann MRI grade scores indicated the extent of disc degeneration, and were markedly lower in the melatonin group compared to the IDD group at 4 and 8 weeks (Figure 7D). The therapeutic effects of melatonin were further confirmed by H&E staining and Safrain O staining (Figure 7B&C). The volume of NP tissue in the control discs was large and occupied most of the space in the discs. The surrounding AF was well organized with its lamellar sheets of collagen. The cells in NP were stellar‐shaped and equably dispersed among the abundant proteoglycan matrix. Compared to the control group, the NP tissue in the IDD group demonstrated reduced size, and was surrounded by disorganized AF. The cells were assembled into clusters, and divided by striped proteoglycan matrix, indicating serious degeneration of NP cells. Melatonin treatment significantly alleviated morphological changes of NP cells and attenuated disorganization and fibrosis of disc structure compared to the IDD group. As shown in the Safrain O staining, the NP tissue in the control group showed positive red staining, indicating a large amount of proteoglycan matrix. The IDD group showed massive proteoglycan loss, whereas treatment of melatonin exhibited less proteoglycan loss compared to the IDD group. In addition, the histological grades of Mel group were both better than the IDD group that evaluated at week 4 and week 8 (Figure 7E). Therefore, these results suggested that melatonin is a potential therapy for IDD.

## 4 DISCUSSION

This study has demonstrated that melatonin treatment can induce Parkin‐mediated mitophagy in NP cells, which cleared damaged mitochondria, reduced the release of ROS and apoptosis factors, and subsequently inhibited cell apoptosis and ECM degeneration induced by oxidative stress. Furthermore, the in vivo study indicated that melatonin may play a protective role in IDD in a puncture‐induced rat model.

Numerous in vivo studies have confirmed the presence of excessive ROS production in aged and degenerated intervertebral discs.^33^ Mitochondrion is not only a primary attack target of ROS, but also a major site of ROS generation.^34^ This vicious cycle can lead to the production of free radicals and mitochondrial dysfunction. Studies have shown that pathogenic factors (for example, mechanical loading, inflammatory cytokines and high glucose) can induce apoptosis by reactive oxygen species‐mediated mitochondrial dysfunction.^12^, ^35^, ^36^ In this study, the administration of TBHP was demonstrated to cause mitochondrial dysfunction and significantly increase the apoptosis of NP cells.

Basal level of mitophagy is essential for sustaining cellular homoeostasis and defending cells against the accrual of dysfunctional mitochondria. During cellular stress, mitophagy and apoptosis activate, causing enhanced mitophagy as an early response to promote survival by removing damaged mitochondria.^17^ However, excessive increases in apoptotic proteases can inhibit the induction of mitophagy, resulting in the accumulation of damaged mitochondria that can lead to cell death.^37^ In recent years, accumulating studies have demonstrated that moderate mitophagy induction exhibits beneficial effects against various diseases, including atherosclerosis,^38^ acute kidney injury,^39^ and traumatic brain injury.^28^ However, the exact molecular mechanisms mediating selective autophagy remain unclear. It has been reported that at least two mechanisms are involved in the regulation of mitophagy in mammal cells, including PINK1/Parkin pathway and NIX/BNIP3L pathway.^40^ The serine/threonine kinase PINK1 is essential for relaying the collapse of the mitochondrial membrane potential to Parkin. PINK1 is located at a low level on mitochondria that have an intact mitochondrial membrane potential, and is cleaved by mitochondrial proteases. Particles are then degraded by proteasomes.^41^ Upon breakdown of the mitochondrial membrane potential (Δψm), the degradation of PINK1 is blocked, and PINK1 can amass on the outer mitochondrial membrane.^42^ The E3 ubiquitin ligase Parkin is predominantly cytosolic under basal conditions, and can be rapidly recruited to mitochondria by PINK1 upon loss of the Δψm.^43^ Parkin then ubiquitinates mitochondrial proteins, which serve as a signal for mitophagy. In this study, we found that TBHP treatment could upregulate PINK1 and downregulate Parkin in NP cells, whereas pretreatment of melatonin could reverse the change of Parkin, instead of PINK1. Interestingly, it has been reported that elevated oxidative stress could block mitophagy by the modification and inactivation of Parkin.^44^, ^45^ Consistent with these studies, in our study, TBHP treatment not only inhibited basal levels of mitophagy, but also reversed melatonin‐induced mitophagy activation via regulation of Parkin activity in NP cells.

To date, the intrinsic and extrinsic apoptosis cascades have been well researched. The intrinsic pathway is initiated by cellular stress and directly affects permeability of the outer mitochondrial membrane via pro‐apoptotic Bcl‐2 family members, including Bax and Bak, leading to the ejection of Cytochrome C.^46^, ^47^ The extrinsic pathway is engaged by a ligation of death receptors and ligands, resulting in adaptor molecule assemblage and pro‐caspase‐8 activation.^48^ Both pathways activate executioner caspases‐3 and −7, which can lead to cell death. To determine the molecular mechanisms involved in melatonin against cellular apoptosis, the levels of intrinsic pathway related proteins, intracellular ROS, mitochondrial membrane potential (Δψm) and ATP levels were investigated after TBHP administration. Our results demonstrated that Bax, Cytochrome C and Cleaved‐caspase3 expression were upregulated by the administration of TBHP, while the levels of Bcl‐2 were down‐regulated. Moreover, TBHP treatment increased intracellular ROS production and encouraged mitochondrial dysfunction by the Δψm collapse, and reduced ATP levels, leading to related protein release and cell apoptosis, while melatonin pretreatment attenuated these effects and improved cell apoptosis. Therefore, these data indicated that the protective role of melatonin against NP cell apoptosis was connected to the inhibited intrinsic apoptosis pathway.

ECM consists of aggrecan proteoglycan and type II collagen, and plays an essential role in the physiological function and maintenance of discs.^49^ ECM metabolism is associated with the redox state of discs.^4^ Several studies have shown that ROS overproduction induced by proinflammatory cytokines or high oxygen tension prominently suppressed ECM synthesis and increased ECM degradation via up‐regulation of matrix degradation proteases in human and rat disc cells.^15^, ^50^, ^51^ Our results showed that melatonin recovered ECM components (for example, type II collagen and aggrecan) via inhibiting the catabolism of ECM components by down‐regulating matrix‐degradation enzymes MMP‐13 and ADAMTS‐5, which was beneficial for maintaining NP homeostasis.

To reveal the relationship between mitophagy, mitochondrial dysfunction and cell apoptosis, a classical mitophagy inhibitor CsA was applied in our study. We found that these beneficial effects disappeared when melatonin‐induced activation of mitophagy was blocked, suggesting that the protective effects of melatonin were mediated by mitophagy stimulation.

Several interesting chemicals exhibit protective effects on disc cells in vitro, but few can translate to clinical therapy for IDD. These chemicals cannot easily disperse into the discs of living animals given the special structure of IVD as the largest avascular organ in the body.^52^ Melatonin is soluble in both aqueous and organic phases, which makes it capable for permeating through the cartilaginous endplate to function on NP cells. To further investigate the therapeutic effects of melatonin in vivo, needle puncture‐induced degenerative rat discs were assessed. Our results showed that significantly degenerated intervertebral discs were observed in the IDD group, and discs with insignificant degeneration were observed in the melatonin group, suggesting that melatonin can mitigate disc degeneration in vivo.

There are several limitations associated with our study. Firstly, although we found that melatonin could enhance mitophagy through the Parkin‐mediated pathway, the exact mechanisms of how melatonin affects Parkin are still unknown, and require further study. Secondly, the relationship between melatonin‐induced mitophagy and inhibition of ECM degradation in NP cells remains unclear. Thirdly, the best delivery route of melatonin and the optimal dose for animals still needs to be further evaluated. Moreover, the in vitro potential protective mechanisms of melatonin by mitophagy induction and cell apoptosis inhibition were not reproduced in vivo*.*

In summary, this study has demonstrated that melatonin treatment can induce mitophagy activation in a Parkin dependent manner in NP cells, which conferred against oxidative stress‐caused cell apoptosis and ECM degradation. Inhibition of mitophagy by an inhibitor (CsA) abolished these desired effects. These findings suggest the potential therapy of melatonin for prevention of disc degeneration.

## CONFLICT OF INTEREST

The authors confirm that there are no conflicts of interest.
